# Supplementary material for: Resolving the full spectrum of human genome variation using Linked-Reads
Source: Genome Res. 2019 Apr;29(4):635–45. doi: 10.1101/gr.234443.118 (PMC6442396; doi:10.1101/gr.234443.118)
Supplement: Supplemental Material [file supp_gr.234443.118_Supplemental_longranger-2.2.zip › longranger-2.2.tar.bz2!/lr2.2-public/lib/go/src/code.google.com/vitess/third_party/py/mock-1.0.1.tar.gz!/mock-1.0.1/html/mock.html]

The Mock Class — Mock 1.0.1 documentation


### Navigation

- index
- next |
- previous |
- Mock 1.0.1 documentation »

# The Mock Class¶

Mock is a flexible mock object intended to replace the use of stubs and
test doubles throughout your code. Mocks are callable and create attributes as
new mocks when you access them [1]. Accessing the same attribute will always
return the same mock. Mocks record how you use them, allowing you to make
assertions about what your code has done to them.

MagicMock is a subclass of Mock with all the magic methods
pre-created and ready to use. There are also non-callable variants, useful
when you are mocking out objects that aren’t callable:
NonCallableMock and NonCallableMagicMock

The patch() decorators makes it easy to temporarily replace classes
in a particular module with a Mock object. By default patch will create
a MagicMock for you. You can specify an alternative class of Mock using
the new\_callable argument to patch.

*class* Mock(*spec=None*, *side\_effect=None*, *return\_value=DEFAULT*, *wraps=None*, *name=None*, *spec\_set=None*, *\*\*kwargs*)¶
:   Create a new Mock object. Mock takes several optional arguments
    that specify the behaviour of the Mock object:

    - spec: This can be either a list of strings or an existing object (a
      class or instance) that acts as the specification for the mock object. If
      you pass in an object then a list of strings is formed by calling dir on
      the object (excluding unsupported magic attributes and methods).
      Accessing any attribute not in this list will raise an AttributeError.

      If spec is an object (rather than a list of strings) then
      \_\_class\_\_ returns the class of the spec object. This allows mocks
      to pass isinstance tests.
    - spec\_set: A stricter variant of spec. If used, attempting to *set*
      or get an attribute on the mock that isn’t on the object passed as
      spec\_set will raise an AttributeError.
    - side\_effect: A function to be called whenever the Mock is called. See
      the side\_effect attribute. Useful for raising exceptions or
      dynamically changing return values. The function is called with the same
      arguments as the mock, and unless it returns DEFAULT, the return
      value of this function is used as the return value.

      Alternatively side\_effect can be an exception class or instance. In
      this case the exception will be raised when the mock is called.

      If side\_effect is an iterable then each call to the mock will return
      the next value from the iterable. If any of the members of the iterable
      are exceptions they will be raised instead of returned.

      A side\_effect can be cleared by setting it to None.
    - return\_value: The value returned when the mock is called. By default
      this is a new Mock (created on first access). See the
      return\_value attribute.
    - wraps: Item for the mock object to wrap. If wraps is not None then
      calling the Mock will pass the call through to the wrapped object
      (returning the real result and ignoring return\_value). Attribute access
      on the mock will return a Mock object that wraps the corresponding
      attribute of the wrapped object (so attempting to access an attribute
      that doesn’t exist will raise an AttributeError).

      If the mock has an explicit return\_value set then calls are not passed
      to the wrapped object and the return\_value is returned instead.
    - name: If the mock has a name then it will be used in the repr of the
      mock. This can be useful for debugging. The name is propagated to child
      mocks.

    Mocks can also be called with arbitrary keyword arguments. These will be
    used to set attributes on the mock after it is created. See the
    configure\_mock() method for details.

    assert\_called\_with(*\*args*, *\*\*kwargs*)¶
    :   This method is a convenient way of asserting that calls are made in a
        particular way:

        ```
        >>> mock = Mock()
        >>> mock.method(1, 2, 3, test='wow')
        <Mock name='mock.method()' id='...'>
        >>> mock.method.assert_called_with(1, 2, 3, test='wow')
        ```

    assert\_called\_once\_with(*\*args*, *\*\*kwargs*)¶
    :   Assert that the mock was called exactly once and with the specified
        arguments.

        ```
        >>> mock = Mock(return_value=None)
        >>> mock('foo', bar='baz')
        >>> mock.assert_called_once_with('foo', bar='baz')
        >>> mock('foo', bar='baz')
        >>> mock.assert_called_once_with('foo', bar='baz')
        Traceback (most recent call last):
          ...
        AssertionError: Expected to be called once. Called 2 times.
        ```

    assert\_any\_call(*\*args*, *\*\*kwargs*)¶
    :   assert the mock has been called with the specified arguments.

        The assert passes if the mock has *ever* been called, unlike
        assert\_called\_with() and assert\_called\_once\_with() that
        only pass if the call is the most recent one.

        ```
        >>> mock = Mock(return_value=None)
        >>> mock(1, 2, arg='thing')
        >>> mock('some', 'thing', 'else')
        >>> mock.assert_any_call(1, 2, arg='thing')
        ```

    assert\_has\_calls(*calls*, *any\_order=False*)¶
    :   assert the mock has been called with the specified calls.
        The mock\_calls list is checked for the calls.

        If any\_order is False (the default) then the calls must be
        sequential. There can be extra calls before or after the
        specified calls.

        If any\_order is True then the calls can be in any order, but
        they must all appear in mock\_calls.

        ```
        >>> mock = Mock(return_value=None)
        >>> mock(1)
        >>> mock(2)
        >>> mock(3)
        >>> mock(4)
        >>> calls = [call(2), call(3)]
        >>> mock.assert_has_calls(calls)
        >>> calls = [call(4), call(2), call(3)]
        >>> mock.assert_has_calls(calls, any_order=True)
        ```

    reset\_mock()¶
    :   The reset\_mock method resets all the call attributes on a mock object:

        ```
        >>> mock = Mock(return_value=None)
        >>> mock('hello')
        >>> mock.called
        True
        >>> mock.reset_mock()
        >>> mock.called
        False
        ```

        This can be useful where you want to make a series of assertions that
        reuse the same object. Note that reset\_mock *doesn’t* clear the
        return value, side\_effect or any child attributes you have
        set using normal assignment. Child mocks and the return value mock
        (if any) are reset as well.

    mock\_add\_spec(*spec*, *spec\_set=False*)¶
    :   Add a spec to a mock. spec can either be an object or a
        list of strings. Only attributes on the spec can be fetched as
        attributes from the mock.

        If spec\_set is True then only attributes on the spec can be set.

    attach\_mock(*mock*, *attribute*)¶
    :   Attach a mock as an attribute of this one, replacing its name and
        parent. Calls to the attached mock will be recorded in the
        method\_calls and mock\_calls attributes of this one.

    configure\_mock(*\*\*kwargs*)¶
    :   Set attributes on the mock through keyword arguments.

        Attributes plus return values and side effects can be set on child
        mocks using standard dot notation and unpacking a dictionary in the
        method call:

        ```
        >>> mock = Mock()
        >>> attrs = {'method.return_value': 3, 'other.side_effect': KeyError}
        >>> mock.configure_mock(**attrs)
        >>> mock.method()
        3
        >>> mock.other()
        Traceback (most recent call last):
          ...
        KeyError
        ```

        The same thing can be achieved in the constructor call to mocks:

        ```
        >>> attrs = {'method.return_value': 3, 'other.side_effect': KeyError}
        >>> mock = Mock(some_attribute='eggs', **attrs)
        >>> mock.some_attribute
        'eggs'
        >>> mock.method()
        3
        >>> mock.other()
        Traceback (most recent call last):
          ...
        KeyError
        ```

        configure\_mock exists to make it easier to do configuration
        after the mock has been created.

    \_\_dir\_\_()¶
    :   Mock objects limit the results of dir(some\_mock) to useful results.
        For mocks with a spec this includes all the permitted attributes
        for the mock.

        See FILTER\_DIR for what this filtering does, and how to
        switch it off.

    \_get\_child\_mock(*\*\*kw*)¶
    :   Create the child mocks for attributes and return value.
        By default child mocks will be the same type as the parent.
        Subclasses of Mock may want to override this to customize the way
        child mocks are made.

        For non-callable mocks the callable variant will be used (rather than
        any custom subclass).

    called¶
    :   A boolean representing whether or not the mock object has been called:

        ```
        >>> mock = Mock(return_value=None)
        >>> mock.called
        False
        >>> mock()
        >>> mock.called
        True
        ```

    call\_count¶
    :   An integer telling you how many times the mock object has been called:

        ```
        >>> mock = Mock(return_value=None)
        >>> mock.call_count
        0
        >>> mock()
        >>> mock()
        >>> mock.call_count
        2
        ```

    return\_value¶
    :   Set this to configure the value returned by calling the mock:

        ```
        >>> mock = Mock()
        >>> mock.return_value = 'fish'
        >>> mock()
        'fish'
        ```

        The default return value is a mock object and you can configure it in
        the normal way:

        ```
        >>> mock = Mock()
        >>> mock.return_value.attribute = sentinel.Attribute
        >>> mock.return_value()
        <Mock name='mock()()' id='...'>
        >>> mock.return_value.assert_called_with()
        ```

        return\_value can also be set in the constructor:

        ```
        >>> mock = Mock(return_value=3)
        >>> mock.return_value
        3
        >>> mock()
        3
        ```

    side\_effect¶
    :   This can either be a function to be called when the mock is called,
        or an exception (class or instance) to be raised.

        If you pass in a function it will be called with same arguments as the
        mock and unless the function returns the DEFAULT singleton the
        call to the mock will then return whatever the function returns. If the
        function returns DEFAULT then the mock will return its normal
        value (from the return\_value.

        An example of a mock that raises an exception (to test exception
        handling of an API):

        ```
        >>> mock = Mock()
        >>> mock.side_effect = Exception('Boom!')
        >>> mock()
        Traceback (most recent call last):
          ...
        Exception: Boom!
        ```

        Using side\_effect to return a sequence of values:

        ```
        >>> mock = Mock()
        >>> mock.side_effect = [3, 2, 1]
        >>> mock(), mock(), mock()
        (3, 2, 1)
        ```

        The side\_effect function is called with the same arguments as the
        mock (so it is wise for it to take arbitrary args and keyword
        arguments) and whatever it returns is used as the return value for
        the call. The exception is if side\_effect returns DEFAULT,
        in which case the normal return\_value is used.

        ```
        >>> mock = Mock(return_value=3)
        >>> def side_effect(*args, **kwargs):
        ...     return DEFAULT
        ...
        >>> mock.side_effect = side_effect
        >>> mock()
        3
        ```

        side\_effect can be set in the constructor. Here’s an example that
        adds one to the value the mock is called with and returns it:

        ```
        >>> side_effect = lambda value: value + 1
        >>> mock = Mock(side_effect=side_effect)
        >>> mock(3)
        4
        >>> mock(-8)
        -7
        ```

        Setting side\_effect to None clears it:

        ```
        >>> from mock import Mock
        >>> m = Mock(side_effect=KeyError, return_value=3)
        >>> m()
        Traceback (most recent call last):
         ...
        KeyError
        >>> m.side_effect = None
        >>> m()
        3
        ```

    call\_args¶
    :   This is either None (if the mock hasn’t been called), or the
        arguments that the mock was last called with. This will be in the
        form of a tuple: the first member is any ordered arguments the mock
        was called with (or an empty tuple) and the second member is any
        keyword arguments (or an empty dictionary).

        ```
        >>> mock = Mock(return_value=None)
        >>> print mock.call_args
        None
        >>> mock()
        >>> mock.call_args
        call()
        >>> mock.call_args == ()
        True
        >>> mock(3, 4)
        >>> mock.call_args
        call(3, 4)
        >>> mock.call_args == ((3, 4),)
        True
        >>> mock(3, 4, 5, key='fish', next='w00t!')
        >>> mock.call_args
        call(3, 4, 5, key='fish', next='w00t!')
        ```

        call\_args, along with members of the lists call\_args\_list,
        method\_calls and mock\_calls are call objects.
        These are tuples, so they can be unpacked to get at the individual
        arguments and make more complex assertions. See
        *calls as tuples*.

    call\_args\_list¶
    :   This is a list of all the calls made to the mock object in sequence
        (so the length of the list is the number of times it has been
        called). Before any calls have been made it is an empty list. The
        call object can be used for conveniently constructing lists of
        calls to compare with call\_args\_list.

        ```
        >>> mock = Mock(return_value=None)
        >>> mock()
        >>> mock(3, 4)
        >>> mock(key='fish', next='w00t!')
        >>> mock.call_args_list
        [call(), call(3, 4), call(key='fish', next='w00t!')]
        >>> expected = [(), ((3, 4),), ({'key': 'fish', 'next': 'w00t!'},)]
        >>> mock.call_args_list == expected
        True
        ```

        Members of call\_args\_list are call objects. These can be
        unpacked as tuples to get at the individual arguments. See
        *calls as tuples*.

    method\_calls¶
    :   As well as tracking calls to themselves, mocks also track calls to
        methods and attributes, and *their* methods and attributes:

        ```
        >>> mock = Mock()
        >>> mock.method()
        <Mock name='mock.method()' id='...'>
        >>> mock.property.method.attribute()
        <Mock name='mock.property.method.attribute()' id='...'>
        >>> mock.method_calls
        [call.method(), call.property.method.attribute()]
        ```

        Members of method\_calls are call objects. These can be
        unpacked as tuples to get at the individual arguments. See
        *calls as tuples*.

    mock\_calls¶
    :   mock\_calls records *all* calls to the mock object, its methods, magic
        methods *and* return value mocks.

        ```
        >>> mock = MagicMock()
        >>> result = mock(1, 2, 3)
        >>> mock.first(a=3)
        <MagicMock name='mock.first()' id='...'>
        >>> mock.second()
        <MagicMock name='mock.second()' id='...'>
        >>> int(mock)
        1
        >>> result(1)
        <MagicMock name='mock()()' id='...'>
        >>> expected = [call(1, 2, 3), call.first(a=3), call.second(),
        ... call.__int__(), call()(1)]
        >>> mock.mock_calls == expected
        True
        ```

        Members of mock\_calls are call objects. These can be
        unpacked as tuples to get at the individual arguments. See
        *calls as tuples*.

    \_\_class\_\_¶
    :   Normally the \_\_class\_\_ attribute of an object will return its type.
        For a mock object with a spec \_\_class\_\_ returns the spec class
        instead. This allows mock objects to pass isinstance tests for the
        object they are replacing / masquerading as:

        ```
        >>> mock = Mock(spec=3)
        >>> isinstance(mock, int)
        True
        ```

        \_\_class\_\_ is assignable to, this allows a mock to pass an
        isinstance check without forcing you to use a spec:

        ```
        >>> mock = Mock()
        >>> mock.__class__ = dict
        >>> isinstance(mock, dict)
        True
        ```

*class* NonCallableMock(*spec=None*, *wraps=None*, *name=None*, *spec\_set=None*, *\*\*kwargs*)¶
:   A non-callable version of Mock. The constructor parameters have the same
    meaning of Mock, with the exception of return\_value and side\_effect
    which have no meaning on a non-callable mock.

Mock objects that use a class or an instance as a spec or spec\_set are able
to pass isintance tests:

```
>>> mock = Mock(spec=SomeClass)
>>> isinstance(mock, SomeClass)
True
>>> mock = Mock(spec_set=SomeClass())
>>> isinstance(mock, SomeClass)
True
```

The Mock classes have support for mocking magic methods. See *magic
methods* for the full details.

The mock classes and the patch() decorators all take arbitrary keyword
arguments for configuration. For the patch decorators the keywords are
passed to the constructor of the mock being created. The keyword arguments
are for configuring attributes of the mock:

```
>>> m = MagicMock(attribute=3, other='fish')
>>> m.attribute
3
>>> m.other
'fish'
```

The return value and side effect of child mocks can be set in the same way,
using dotted notation. As you can’t use dotted names directly in a call you
have to create a dictionary and unpack it using \*\*:

```
>>> attrs = {'method.return_value': 3, 'other.side_effect': KeyError}
>>> mock = Mock(some_attribute='eggs', **attrs)
>>> mock.some_attribute
'eggs'
>>> mock.method()
3
>>> mock.other()
Traceback (most recent call last):
  ...
KeyError
```

*class* PropertyMock(*\*args*, *\*\*kwargs*)¶
:   A mock intended to be used as a property, or other descriptor, on a class.
    PropertyMock provides \_\_get\_\_ and \_\_set\_\_ methods so you can specify
    a return value when it is fetched.

    Fetching a PropertyMock instance from an object calls the mock, with
    no args. Setting it calls the mock with the value being set.

    ```
    >>> class Foo(object):
    ...     @property
    ...     def foo(self):
    ...         return 'something'
    ...     @foo.setter
    ...     def foo(self, value):
    ...         pass
    ...
    >>> with patch('__main__.Foo.foo', new_callable=PropertyMock) as mock_foo:
    ...     mock_foo.return_value = 'mockity-mock'
    ...     this_foo = Foo()
    ...     print this_foo.foo
    ...     this_foo.foo = 6
    ...
    mockity-mock
    >>> mock_foo.mock_calls
    [call(), call(6)]
    ```

Because of the way mock attributes are stored you can’t directly attach a
PropertyMock to a mock object. Instead you can attach it to the mock type
object:

```
>>> m = MagicMock()
>>> p = PropertyMock(return_value=3)
>>> type(m).foo = p
>>> m.foo
3
>>> p.assert_called_once_with()
```

# Calling¶

Mock objects are callable. The call will return the value set as the
return\_value attribute. The default return value is a new Mock
object; it is created the first time the return value is accessed (either
explicitly or by calling the Mock) - but it is stored and the same one
returned each time.

Calls made to the object will be recorded in the attributes
like call\_args and call\_args\_list.

If side\_effect is set then it will be called after the call has
been recorded, so if side\_effect raises an exception the call is still
recorded.

The simplest way to make a mock raise an exception when called is to make
side\_effect an exception class or instance:

```
>>> m = MagicMock(side_effect=IndexError)
>>> m(1, 2, 3)
Traceback (most recent call last):
  ...
IndexError
>>> m.mock_calls
[call(1, 2, 3)]
>>> m.side_effect = KeyError('Bang!')
>>> m('two', 'three', 'four')
Traceback (most recent call last):
  ...
KeyError: 'Bang!'
>>> m.mock_calls
[call(1, 2, 3), call('two', 'three', 'four')]
```

If side\_effect is a function then whatever that function returns is what
calls to the mock return. The side\_effect function is called with the
same arguments as the mock. This allows you to vary the return value of the
call dynamically, based on the input:

```
>>> def side_effect(value):
...     return value + 1
...
>>> m = MagicMock(side_effect=side_effect)
>>> m(1)
2
>>> m(2)
3
>>> m.mock_calls
[call(1), call(2)]
```

If you want the mock to still return the default return value (a new mock), or
any set return value, then there are two ways of doing this. Either return
mock.return\_value from inside side\_effect, or return DEFAULT:

```
>>> m = MagicMock()
>>> def side_effect(*args, **kwargs):
...     return m.return_value
...
>>> m.side_effect = side_effect
>>> m.return_value = 3
>>> m()
3
>>> def side_effect(*args, **kwargs):
...     return DEFAULT
...
>>> m.side_effect = side_effect
>>> m()
3
```

To remove a side\_effect, and return to the default behaviour, set the
side\_effect to None:

```
>>> m = MagicMock(return_value=6)
>>> def side_effect(*args, **kwargs):
...     return 3
...
>>> m.side_effect = side_effect
>>> m()
3
>>> m.side_effect = None
>>> m()
6
```

The side\_effect can also be any iterable object. Repeated calls to the mock
will return values from the iterable (until the iterable is exhausted and
a StopIteration is raised):

```
>>> m = MagicMock(side_effect=[1, 2, 3])
>>> m()
1
>>> m()
2
>>> m()
3
>>> m()
Traceback (most recent call last):
  ...
StopIteration
```

If any members of the iterable are exceptions they will be raised instead of
returned:

```
>>> iterable = (33, ValueError, 66)
>>> m = MagicMock(side_effect=iterable)
>>> m()
33
>>> m()
Traceback (most recent call last):
 ...
ValueError
>>> m()
66
```

# Deleting Attributes¶

Mock objects create attributes on demand. This allows them to pretend to be
objects of any type.

You may want a mock object to return False to a hasattr call, or raise an
AttributeError when an attribute is fetched. You can do this by providing
an object as a spec for a mock, but that isn’t always convenient.

You “block” attributes by deleting them. Once deleted, accessing an attribute
will raise an AttributeError.

```
>>> mock = MagicMock()
>>> hasattr(mock, 'm')
True
>>> del mock.m
>>> hasattr(mock, 'm')
False
>>> del mock.f
>>> mock.f
Traceback (most recent call last):
    ...
AttributeError: f
```

# Attaching Mocks as Attributes¶

When you attach a mock as an attribute of another mock (or as the return
value) it becomes a “child” of that mock. Calls to the child are recorded in
the method\_calls and mock\_calls attributes of the
parent. This is useful for configuring child mocks and then attaching them to
the parent, or for attaching mocks to a parent that records all calls to the
children and allows you to make assertions about the order of calls between
mocks:

```
>>> parent = MagicMock()
>>> child1 = MagicMock(return_value=None)
>>> child2 = MagicMock(return_value=None)
>>> parent.child1 = child1
>>> parent.child2 = child2
>>> child1(1)
>>> child2(2)
>>> parent.mock_calls
[call.child1(1), call.child2(2)]
```

The exception to this is if the mock has a name. This allows you to prevent
the “parenting” if for some reason you don’t want it to happen.

```
>>> mock = MagicMock()
>>> not_a_child = MagicMock(name='not-a-child')
>>> mock.attribute = not_a_child
>>> mock.attribute()
<MagicMock name='not-a-child()' id='...'>
>>> mock.mock_calls
[]
```

Mocks created for you by patch() are automatically given names. To
attach mocks that have names to a parent you use the attach\_mock()
method:

```
>>> thing1 = object()
>>> thing2 = object()
>>> parent = MagicMock()
>>> with patch('__main__.thing1', return_value=None) as child1:
...     with patch('__main__.thing2', return_value=None) as child2:
...         parent.attach_mock(child1, 'child1')
...         parent.attach_mock(child2, 'child2')
...         child1('one')
...         child2('two')
...
>>> parent.mock_calls
[call.child1('one'), call.child2('two')]
```

---

|  |  |
| --- | --- |
| [1] | The only exceptions are magic methods and attributes (those that have leading and trailing double underscores). Mock doesn’t create these but instead of raises an AttributeError. This is because the interpreter will often implicitly request these methods, and gets *very* confused to get a new Mock object when it expects a magic method. If you need magic method support see *magic methods*. |

### Table Of Contents

- The Mock Class
- Calling
- Deleting Attributes
- Attaching Mocks as Attributes

#### Previous topic

Mock - Mocking and Testing Library

#### Next topic

Patch Decorators

### This Page

- Show Source

### Quick search


Enter search terms or a module, class or function name.

### Navigation

- index
- next |
- previous |
- Mock 1.0.1 documentation »

© Copyright 2007-2012, Michael Foord & the mock team.
Last updated on Nov 05, 2012.
Created using Sphinx 1.1.3.
